# Fourier analysis of vertical forces to integrate balance measurements

**DOI:** 10.1186/1757-1146-7-S1-A23

**Published:** 2014-04-08

**Authors:** Claudia Giacomozzi, Francesco Martelli, Massimo Lillia, Antonello Fadda

**Affiliations:** 1 Department of Technology and Health, Istituto Superiore di Sanità, Rome, Italy; 2 Electronic High School ISIS Arturo Malignani, Udine, Italy

## Background

Balance during orthostatic standing is usually investigated through force platforms and consolidated sets of parameters and protocols [1] based on 51.2s of observation period and on analysis of centre of pressure (COP) movements in the time and in the frequency domain. Force instantaneous changes associated to centre of mass vertical oscillations had been scarcely investigated [2], maybe due to inadequate signal quality, even though they may represent an added value to gain knowledge in muscular strategies adopted to maintain balance under pathological conditions. This study aims at assessing a newly arranged force plate to obtain a reliable insight into the vertical force frequency spectrum; two small samples are used to evaluate feasibility and relevance, i.e. a normal active healthy population (controls) and trained athletes.

## Materials and methods

The PODATA force plate (GPS400, Chinesport, Udine, Italy) has been used, which integrates traditional optical podoscopy with dynamometry. The device relies on 6 calibrated uniaxial load cells which deliver a highly accurate dataset in terms of instantaneous COP coordinates and vertical force value (12bit A/D converter; overall resolution 0.0125 kg, linear accuracy 5%, angular accuracy 2.5%, sampling rate 200 Hz). The observation period is limited to 20s since Manufacturer pre-market clinical investigations had indicated this as the longest observation window during which healthy individuals successfully recover the initial COP position. Preliminary accuracy tests were performed by using a purposely designed physical pendulum whose mass distribution and size determined a full oscillation period of 2.5s, thus entailing the fundamental frequency of 0.4Hz for COP, doubled to 0.8Hz for vertical force. Then, two groups of 5 controls (41±6 years; 68±18 kg) and 5 trained judoka athletes (37±6 years; 80±10 kg) were examined during open-eye orthostatic standing. COP and vertical force frequency spectra were obtained by applying a rectangular FFT over the acquired 4096 samples (exact observation time 20.48s, frequency resolution 0.05Hz).

## Results

**Figure 1:**
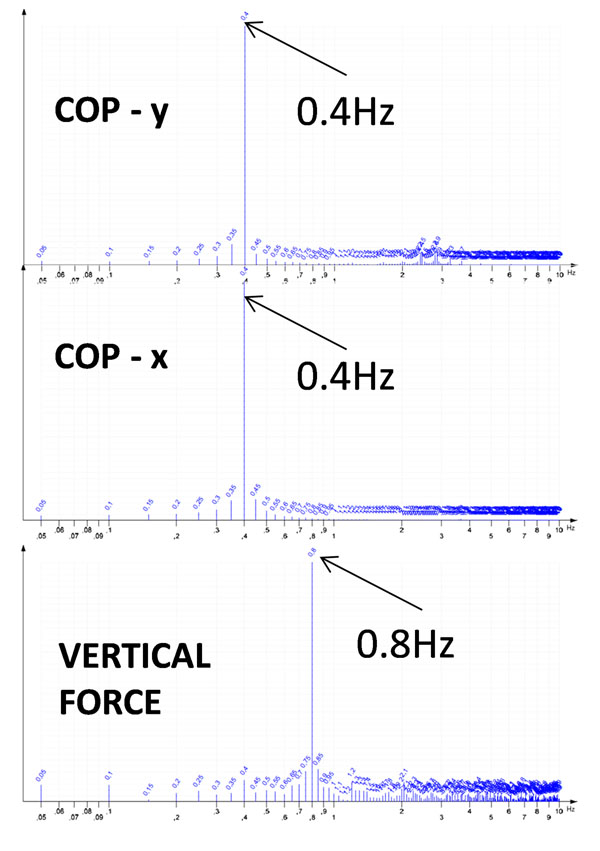
Test with physical pendulum (24.2kg, oscillation period 2.5s). Frequency spectra of COP coordinates and Vertical force (Log scale, resolution 0.05Hz)

**Figure 2:**
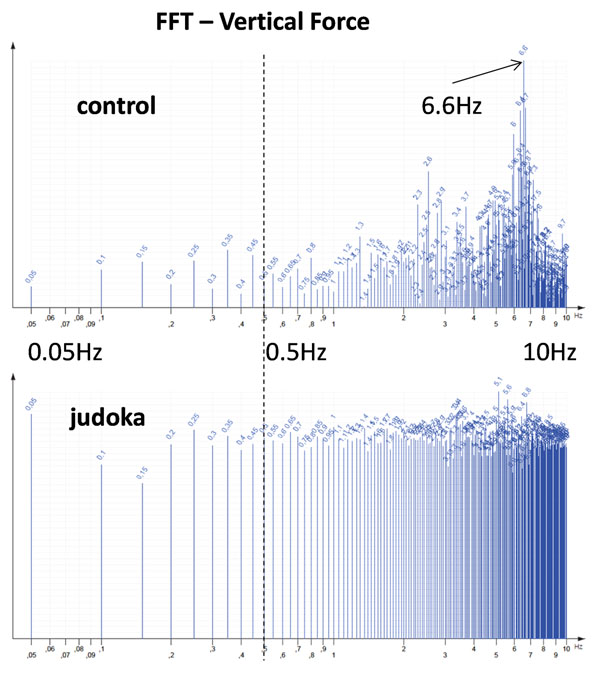
FFT of vertical force from one control and one judoka (Log scale, resolution 0.05Hz)

The preliminary tests with the physical pendulum confirmed the appropriateness of the PODATA system to accurately detect fundamental frequencies (Figure 1). The on-the-field application showed: no statistically significant differences between COP frequency spectra of the two groups; significantly higher vertical force median frequency (5.63±0.24 Hz) of controls with respect to athletes (4.76±0.16 Hz) (Figure 2); significantly lower energy concentrated at very low frequencies in controls (percentiles at 0.5 Hz: 4.01±2.80 (controls), 11.03±1.22 (athletes)); significantly higher % variations of vertical force in athletes (0.14±0.02) than in controls (0.10±0.01).

## Conclusions

The proposed device and protocol proved adequate for an accurate analysis of vertical force in the frequency domain. Peculiar changes in its spectrum, when integrated with those of COP movements, may help in better understanding the model of muscular activation set up to maintain balance, i.e. the physiological inverse pendulum model or rather other complex models originated by pathological conditions.
